# Principles Governing Malleting Force

**Published:** 1897-02

**Authors:** 


### Editorial.

### PRINCIPLES GOVERNING MALLETING FORCE.

It is a somewhat singular fact that although the mallet has been used in dentistry almost from the beginning of the present century and has gone through the various phases of development from the ordinary hammer to the electrical and mechanical mallets invented by Bonwill, yet there remains an amount of misconception—or perhaps it would be better called indifference—in regard to the theory and use of this instrument that is not altogether creditable to those who regard it as all important in operative dentistry.

It is, therefore, with unusual satisfaction that the remarks of Dr. W. St. George Elliott in the proceedings of The New York Institute of Stomatology, present number, were received and read. His statements are very suggestive, and indicate a tendency towards a scientific solution of the question of malleting in its entirety.

It is very true, as Dr. Elliott asserts, that it is next to impossible to change the practice of individuals, for, "If a process were demonstrated for condensing gold, which would meet every requirement, which would be scientific, accurate, and rapid, there would not be a single person who would adopt it, all continuing to do just as they had been doing."

This, unquestionably, has its discouraging side, but the true scientist never allows the whims and conservatism of professional thought to disturb him, for his vocation is to attain the absolute, and, this acquired, it is immaterial whether it be adopted or not, for the certainty remains that the truth established will eventually become the dominant thought.

The history of the mallet in dentistry cannot, it is presumed, be correctly written to-day. Koecker, in 1826, writes of it as "surprising and repugnant that after the tooth is prepared for the reception of the stopping, some operators actually *employ a hammer* and *punch* to drive the metal into the cavity of the tooth."

Fitch, in 1835, also condemns it as a "most injurious manner of introducing and compacting the metal." Dr. Atkinson has generally been given the credit of its introduction, but he deserves to be remembered, in this connection, solely because of his persistency in calling attention to its value in condensing gold.

The necessity for a clearer understanding of this instrument in its relation to filling teeth led the writer many years ago to attempt the investigation of the theory upon which it was used and the comparative value of the various forms of mallets then in use. This effort, while not as complete or as carefully prepared as it might have been, occupied much time, and the results were given in the *Dental Times*, 1871. These are condensed in this article.

The theory of the use of the mallet is that it will compact more thoroughly than any regular force, no matter how long continued. This is illustrated simply by the driving of a nail. Pounds' weight may be applied to a nail by ordinary pressure, and it will fail to penetrate the soft wood; but the application of a small hammer will, by the sudden concussion, implant it without difficulty. When the force is equal, the resisting force and the force applied, the union or condensation of gold will be comparatively perfect. The idea that prevails that an increase of force by a multiplication of weight will impact to a greater extent is only in part true; force and weight impact, but an increase beyond certain limits will fail in giving greater density.

The force of a body in motion, or its momentum, is increased by the velocity; if that be doubled the force is doubled, and so on in regular proportion. If, then, one body be in motion and another in rest, the impact produces a loss of force in the body moving, which force is imparted to the one at rest, so that the force of the two combined equals the original force in the moving object. "In all cases the momentum after impact must be the sum of the momenta before impact, . . . so that the momentum lost by one body, by the collision, is exactly equal to the momentum gained by the other." The third law of Newton expresses it,—"Action and reaction are equal and contrary."

If we have a non-resisting or movable body as a base, it is found that every blow struck loses a certain proportion of its effect. It is a well-known law that an appreciable amount of time is required for a blow to distribute itself throughout the particles of matter of the object struck.

If, then, gold-foil be in process of condensation, and a hard base be used to support the matrix, the "hand-pressure" method will produce a density nearly if not quite equal to that where a heavy mallet is used, but if in place of a solid base a soft substance is used to support the matrix, as a cushion, great loss of condensing power is the result. Velocity overcomes mobility, or susceptibility to motion; weight increases it.

What, then, necessarily follows from these deductions? That velocity on a partially non-resisting body, as a tooth in the mouth, is of vastly more importance than weight. Weight decreases velocity. Reasoning then from this basis, it would seem that the most effective mallet that can be made must be one that combines lightness with solidity. Now, where do we find these qualities to the greatest extent? In the light steel mallet, and they are represented in the least quantity in the lead mallet.

To test the correctness of these views, a number of fillings, twelve gold and four tin, were prepared in a hardened polished steel matrix made in separable parts, so that the fillings could be removed without loss or injury. These were carefully marked and separately weighed on the delicate scales of the United States Mint.

It is unnecessary to give the results in detail, but it may be of interest to repeat the fact that it was demonstrated beyond question that rapidity of blow overcame the tendency of motion in an unstable object, as, for instance, the human body during the process of filling teeth. The four fillings made by Bonwill's electrical mallet, prepared by different operators, varied but little. With the matrix placed on a soft cushion, to represent mobility, two weighed two hundred and ninety and a quarter and two hundred and eighty-seven and a half milligrammes respectively. Two on a hard base weighed two hundred and eighty-seven and two hundred and seventy-two milligrammes, showing a difference in favor of the soft base, or, practically, that velocity of blow absolutely overcame mobility. The half-ounce steel hammer on a hard base did more effectual service than a two-ounce lead mallet by seven and a half milligrammes, and a six-ounce wood mallet on a non-resisting medium, as spunk, was not as effective as hand-pressure on a resisting surface, as wood, there being fifty and one-quarter milligrammes in favor of the latter.

The analysis of this table might be repeated further, but it would not change the result, as, for instance, the slow acting automatic gave but two hundred and seventeen milligrammes of weight on a non-resisting base.

The experiments in part detailed were not made with the view of indorsing any special mallet or hand-pressure, but to endeavor to elucidate the reasons for their use and their relative value as condensing instruments. It would, doubtless, have added much to the value of these experiments had the specific gravity of the resulting fillings been taken, as this certainly is the only true test, but for practical purposes it was then considered sufficient to test by weight upon scales so delicately accurate that no criticism could be made in this direction.

There is one feature of Dr. Elliott's work that seems worthy of attention, and that is the difference in time between the filling placed in by the "man who malleted for himself" and the one who "had an assistant to mallet for him." The first took "an hour and a half to perform the operation," while the latter required "an hour and three-quarters," and those using the "electrics took about five hours each." It is presumed the one malleting for himself had an assistant to hand the gold. This demonstrates, if it demonstrates anything, that the amount of time lost by the operator in picking up gold and, perhaps, annealing is far in excess of the mere packing, and proves the economic value of having an aid in this operation.

It has been too much the custom of some operators to regard as of great importance rapidity in condensing gold. It is certainly important from both the operator's and patient's point of view, but there are so many conditions essential to perfection in a filling that rapidity cannot be considered a question of vital importance in determining the skill of an operator.

The value of the mallet may be regarded as settled in the minds of most operators, and the old method of hand-pressure has largely passed into oblivion. The question of solidity, as an important factor, has also been settled by the judgment of the many as being essential to the preservation of the tooth.

There probably can be no effectual dispute to this self-evident proposition, but the point has never yet been decided, whether severe malleting is better for tooth material than the less forceful pressure of the gold by hand.

Histological workers on human teeth know very well that it is rare to find enamel free from cracks. These can be macroscopically observed, very frequently, upon the labial surfaces of anterior teeth. Every crack so placed in enamel may, if unfavorably situated, become the conduit for fluids and the subsequent pathological changes. That the blows of the mallet add to these fractures there can be no question, and especially must this be true at the cervical border. Hence, in the opinion of the writer, the use of hand-packing along delicate walls should never be dispensed with, whether these walls be lined with cohesive or non-cohesive gold.

The proof of the solidity of a filling is not readily obtained, and it necessarily becomes a mere supposition unless the filling be removed. Dr. Elliott very properly criticises the idea that gold rolled into plate is a certain demonstration of solidity. The most imperfectly condensed filling can be made as solid as gold plate by this process. The writer in some experiments with gold-foil, some years since, placed in a tooth a large filling by the Herbst method, and purposely made it a poorly condensed filling. This was removed, annealed, and rolled. It came out a very perfect plate. A portion of this was taken and given to Abbey & Sons, who made it again into foil. From foil to foil, while interesting as a fact, proved only one thing, that solidity of the gold filling was not an essential factor in the process.

That the mallet, properly used, has been an aid to more perfected operations cannot be disputed, but it would seem that the dental profession has lost sight of the fact that teeth were preserved quite as effectually under the old methods as they are under the excessive hammering of to-day, and it would seem as though more lasting work could be accomplished, with less nervous strain to patients, if present methods could more generally be engrafted upon those of the old masters, who repudiated the mallet and all accessories, supposed to be so essential for good work at the present time.

